# Sotagliflozin and Kidney Outcomes, Kidney Function, and Albuminuria in Type 2 Diabetes and CKD: A Secondary Analysis of the SCORED Trial

**DOI:** 10.2215/CJN.0000000000000414

**Published:** 2024-01-26

**Authors:** Vikas S. Sridhar, Deepak L. Bhatt, Ayodele Odutayo, Michael Szarek, Michael J. Davies, Phillip Banks, Bertram Pitt, Ph. Gabriel Steg, David Z.I. Cherney

**Affiliations:** 1 Division of Nephrology, Department of Medicine, University of Toronto, Toronto, Ontario, Canada; 2 Mount Sinai Fuster Heart Hospital Icahn School of Medicine at Mount Sinai, New York, New York; 3 University of Colorado Anschutz Medical Campus, Aurora, Colorado; 4 CPC Clinical Research, Aurora, Colorado; 5 State University of New York, Downstate Health Sciences University, Brooklyn, New York; 6 Lexicon Pharmaceuticals, Inc., The Woodlands, Texas; 7 University of Michigan, Ann Arbor, Michigan; 8 AP-HP, Hôpital Bichat, INSERM U-1148, Université Paris-Cité, Paris, France

**Keywords:** CKD, diabetes, outcomes, diabetic kidney disease, pharmacology

## Abstract

**Background:**

In the initial analysis of the Effect of Sotagliflozin on Cardiovascular and Renal Events in Patients with Type 2 Diabetes and Moderate Renal Impairment Who Are at Cardiovascular Risk (SCORED) trial, because of early trial termination and suspension of adjudication, reconciliation of eGFR laboratory data and case report forms had not been completed. This resulted in a small number of kidney composite events and a nominal effect of sotagliflozin versus placebo on this outcome. This exploratory analysis uses laboratory eGFR data, regardless of case report form completion, to assess the effects of sotagliflozin on the predefined kidney composite end point in the SCORED trial and additional cardiorenal composite end points.

**Methods:**

SCORED was a multicenter, randomized trial evaluating cardiorenal outcomes with sotagliflozin versus placebo in 10,584 patients with type 2 diabetes and CKD. This exploratory analysis used laboratory data to derive the eGFR components and case report form data for the non-laboratory–defined components that together made up the kidney and cardiorenal composites. AKI was also assessed in this dataset.

**Results:**

Using laboratory data, 223 events were identified, and sotagliflozin reduced the risk of the composite of first event of sustained ≥50% decline in eGFR, eGFR <15 ml/min per 1.73 m^2^, dialysis, or kidney transplant with 87 events (1.6%) in the sotagliflozin group and 136 events (2.6%) in the placebo group (hazard ratio [95% confidence interval], 0.62 [0.48 to 0.82]), *P* < 0.001). Sotagliflozin reduced the risk of a cardiorenal composite end point defined as the abovementioned composite plus cardiovascular or kidney death with 239 events (4.5%) in the sotagliflozin group and 306 events (5.7%) in the placebo group (hazard ratio [95% confidence interval], 0.77 [0.65 to 0.91], *P* = 0.0023). The results were consistent when using different eGFR decline thresholds and when only including kidney death in composites (all *P* < 0.01). The incidence of AKI was similar between treatment groups.

**Conclusions:**

In this exploratory analysis using the complete laboratory dataset, sotagliflozin reduced the risk of kidney and cardiorenal composite end points in patients with type 2 diabetes and CKD.

**Clinical Trial registry name and registration number:**

ClinicalTrials.gov Identifier: NCT03315143.

## Visual Abstract

**Figure:**
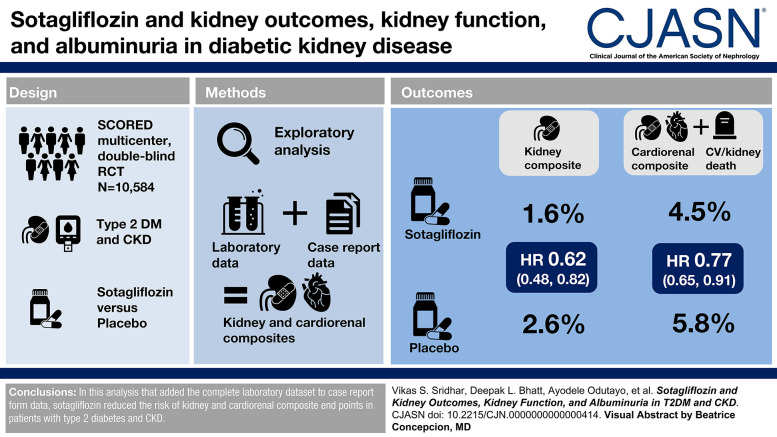

## Introduction

In pivotal clinical trials, sodium glucose cotransporter (SGLT) inhibitors reduce the incidence of clinical end points, including cardiovascular (CV) events, CKD progression, and heart failure.^1^ These risk reductions are consistent irrespective of baseline comorbidities, such as diabetes, heart failure, and CKD, and have resulted in multiple guideline recommendations for the use of SGLT inhibitors for the clinical management of CV disease and CKD.^2,3^

Sotagliflozin is a dual SGLT1 and 2 inhibitor that has demonstrated CV benefits.^4–11^ On the basis of the Effect of Sotagliflozin on Cardiovascular Events in Patients with Type 2 Diabetes Post Worsening Heart Failure and Effect of Sotagliflozin on Cardiovascular and Renal Events in Patients with Type 2 Diabetes and Moderate Renal Impairment Who Are at Cardiovascular Risk (SCORED) trials, sotagliflozin has been approved by the Food and Drug Administration to reduce the risk of CV death, hospitalization for heart failure, and urgent heart failure visits in adults with heart failure or type 2 diabetes, CKD, and other CV risk factors. Beyond cardioprotective benefits, sotagliflozin significantly reduced albuminuria and the risk of progression of albuminuria in patients with type 2 diabetes and CKD.^12^ Moreover, the incidence of the composite kidney end point of a sustained ≥50% decrease in eGFR, sustained eGFR of <15 ml/min per 1.73 m^2^, dialysis, or kidney transplant was reduced by 29% (hazard ratio [HR] [95% confidence interval (CI)], 0.71 [0.46 to 1.08]), albeit not statistically significant in the SCORED trial.^4^ However, because the SCORED trial was stopped early because of lack of funding, the median duration of follow-up was only 16 months, which limited the number of events and statistical power to assess some outcomes, such as CKD progression.

Importantly, changes in eGFR reported in the kidney composite outcome in the primary report from the SCORED trial were determined on the basis of investigator review of laboratory data and reported on a case report form designed to document events related to eGFR decreases. Owing to the early termination of the trial and cessation of adjudication, reconciliation between the case report form data and available laboratory data for the eGFR-based end points was not completed. Therefore, the purpose of this exploratory analysis was to use laboratory eGFR data, regardless of case report form completion, to assess the effects of sotagliflozin on the predefined kidney composite end point in the SCORED trial and additional cardiorenal composite end points. Use of the laboratory data was expected to yield more eGFR-derived events than reported with dedicated case report forms in the original analysis and, therefore, allows a more comprehensive evaluation of cardiorenal outcomes.

## Methods

The complete study methods for the SCORED trial were previously published.^4^ Briefly, SCORED was a multinational, placebo-controlled, double-blind, randomized study of 10,584 patients with type 2 diabetes (hemoglobin A1c [HbA1c] ≥7%), CKD (eGFR 25–60 ml/min per 1.73 m^2^ regardless of urinary albumin–creatinine ratio [UACR]), and higher CV risk. Patients were randomized 1:1 to sotagliflozin (200 mg/d uptitrated to 400 mg/d) versus matching placebo. Ethics committee approval was obtained at all participating institutions, and patients provided written informed consent. Patients were enrolled in 750 sites in 44 countries. The first patient was randomized on December 8, 2017, and the last on January 20, 2020.

Relevant to this analysis, serum creatinine (and calculated eGFR using the Modification of Diet in Renal Disease formula) was assessed from blood draws collected at baseline and postrandomization clinical visits scheduled at weeks 4, 8, and 26 and every 6 months thereafter.^4^

As previously mentioned, the kidney composite end point in the primary report of the SCORED trial was a sustained ≥50% decline from baseline in eGFR or kidney failure defined as an eGFR <15 ml/min per m^2^, kidney transplant, or initiation and maintenance of dialysis.^4^ The eGFR-based components were to have been confirmed for at least 30 days or at the last value. Data for each component end point were to be captured on dedicated case report forms. Because there was no reconciliation between the case report form data and available laboratory data for the eGFR-based kidney component end points and to be consistent with derivation of the primary end point (CV death and heart failure–related events), only dedicated case report form data were used to analyze the abovementioned kidney-specific composite end point.

In contrast to the primary report of the SCORED trial, this exploratory analysis used laboratory data to derive the eGFR components and case report form data for the non-laboratory–defined components (*e.g*., kidney transplant). This approach was expected to yield more eGFR-based events for these analyses and was used to evaluate the predefined kidney composite end point—the primary outcome of this analysis. In addition, cardiorenal and kidney-specific composite outcomes evaluated in previous SGLT2 inhibitor trials were analyzed with the SCORED data. Another focus of this *post hoc* analysis was the cardiorenal composite of first event of ≥50% decline in eGFR, kidney failure, CV death, or kidney death. Additional cardiorenal end points examined in our analysis were (*1*) ≥57% decline in eGFR, kidney failure, CV death, or kidney death^13^; (*2*) ≥40% decline in eGFR, kidney failure, CV death, or kidney death; (*3*) ≥57% decline in eGFR, kidney failure, and kidney death; (*4*) ≥50% decline in eGFR, kidney failure, and kidney death; and (*5*) 40% decline in eGFR, kidney failure, and kidney death.

Subgroup analyses were conducted to assess for effect modification by baseline eGFR (<30, ≥30 to <45, and ≥45 ml/min per 1.73 m^2^), baseline UACR (<30 [normoalbuminuria; Kidney Disease Improving Global Outcomes (KDIGO) A1], ≥30 to <300 [moderately increased albuminuria; KDIGO A2], and ≥300 mg/g [severely increased albuminuria; KDIGO A3]), and baseline HbA1c (<8, ≥8 to <9, and ≥9%).

The incidence of AKI was also assessed in this analysis. Events of AKI were identified using the Acute Renal Failure Standardized MedDRA Queries SMQ 20000003. Data were analyzed overall and by the following baseline subgroup categories (age group, sex, body mass index, eGFR, left ventricular ejection fraction, and region). Incidence (*n* [%]) was tabulated for each treatment and by each subgroup.

### Statistical Analyses

Treatment comparisons for each composite end point were performed by proportional hazards models with stratification by heart failure criteria and geographic region and treatment assignment as the sole predictor. Because subgroups were defined by ordered categories of quantitative variables, each interaction *P* value reflects a test of linear trend in the estimated log HRs across categories from a stratified proportional hazards model containing treatment assignment, the ordered subgroup variable, and their interaction. Cumulative incidence was estimated by Kaplan–Meier methods.

## Results

### Baseline Characteristics

Baseline characteristics and patient disposition for the trial have been reported previously.^4^ For kidney-related measurements, the median eGFR was 45 ml/min per 1.73 m^2^ (interquartile range [IQR], 37–51 ml/min per 1.73 m^2^), with 8%, 44%, and 48% of patients with an eGFR <30, ≥30 to <45, and ≥45 ml/min per 1.73 m^2^, respectively. The median UACR was 75 mg/g (IQR, 17–481 mg/g), with 35%, 34%, and 31% categorized as normoalbuminuria (KDIGO A1), moderately increased albuminuria (KDIGO A2), and severely increased albuminuria (KDIGO A3), respectively (Supplemental Table 1). The median duration of follow-up was 16 months (IQR, 12–20 months) in the sotagliflozin group and 16 months (IQR, 12–20 months) in the placebo group.

### Effects of Sotagliflozin on the Composite Kidney-Specific and Cardiorenal Outcomes

**Figure 1:**
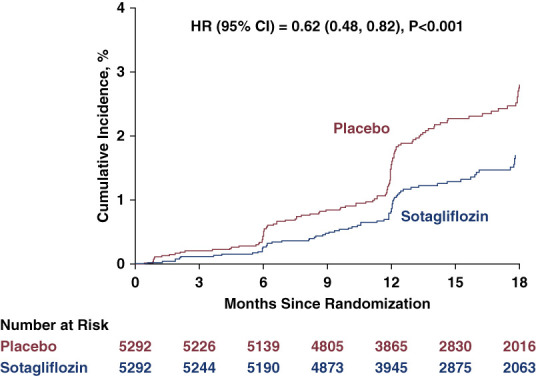
**Cumulative incidence curve for the composite of first event of 50% decline in eGFR, kidney failure, or kidney death in the analysis using laboratory data for eGFR results.** CI, confidence interval; HR, hazard ratio.

For the predefined kidney-specific composite end point (sustained ≥50% decrease in eGFR or kidney failure), only 89 total events were reported in the case report forms, with 37 of 5292 patients (0.7%) in the sotagliflozin group and 52 of 5292 (1%) in the placebo group. The effect of sotagliflozin on this composite was not statistically significant (HR [95% CI], 0.71 [0.46 to 1.08]; *P* = 0.10).^4^ When using the laboratory data for eGFR components, a total of 223 events were identified for the predefined kidney-specific composite end point, with 87 of 5292 patients (2%) in the sotagliflozin group and 136 of 5292 (3%) in the placebo group. Sotagliflozin significantly reduced the risk of this composite by 38% (HR [95% CI], 0.62 [0.48 to 0.82]; *P* < 0.001) (Figure 1 and Table 1).

**Table 1:** Comparison of results for the prespecified kidney-specific composite end point of first event of 50% decline in eGFR or kidney failure defined as eGFR <15 ml/min per 1.73 m^2^, maintenance dialysis, or kidney transplant using the case report form or laboratory data for eGFR results

Kidney Composite and Components	Sotagliflozin (*N*=5292), *n* (%)	Placebo (*N*=5292), *n* (%)	HR (95% CI)	*P* Value
**eGFR determination from case report form (*i.e*., 50% reduction or <15 ml/min per 1.73 m** ^**2**^ **)**				
First event in composite consisting of 50% decline in eGFR (sustained or last value), eGFR <15 ml/min per m^2^ (sustained or last value), maintenance dialysis, or kidney transplant	37 (0.7)	52 (1.0)	0.71 (0.46 to 1.08)	0.10
50% decline in eGFR	23 (0.4)	29 (0.5)	0.79 (0.45 to 1.36)	0.39
Maintenance dialysis or kidney transplant	15 (0.3)	22 (0.4)	0.68 (0.35 to 1.30)	0.24
eGFR <15 ml/min per m^2^	17 (0.3)	26 (0.5)	0.65 (0.35 to 1.19)	0.16
**Using laboratory eGFR for results (*i.e*., 50%** ** reduction or <15 ml/min per 1.73 m**^**2**^**)**				
First event in composite consisting of 50% decline in eGFR (sustained or last value), eGFR <15 ml/min per 1.73 m^2^ (sustained or last value), maintenance dialysis, or kidney transplant	87 (1.6)	136 (2.6)	0.62 (0.48 to 0.82)	<0.001
50% decline in eGFR	75 (1.4)	121 (2.3)	0.60 (0.45 to 0.81)	<0.001
Maintenance dialysis or kidney transplant	15 (0.3)	22 (0.4)	0.67 (0.35 to 1.30)	0.24
eGFR <15 ml/min per m^2^	41 (0.8)	68 (1.3)	0.60 (0.41 to 0.88)	0.009

CI, confidence interval; HR, hazard ratio.

The exploratory cardiorenal composite outcome of the first event of ≥50% decline in eGFR, kidney failure, CV death, or kidney death occurred in 239 of 5292 participants (5%) in the sotagliflozin group and 306 of 5292 participants (6%) in the placebo group (HR, 0.77; 95% CI, 0.65 to 0.91; *P* = 0.002; Table 2). The event rates for individual components of the composite outcome generally favored sotagliflozin versus placebo, albeit with a limited number of kidney deaths and no reported kidney transplants (Table 2). Event curves appeared to diverge around month 3 and progressively separated over the follow-up period (Supplemental Figure 1).

**Table 2:** Summary of composite of first event of 50% decline in eGFR, kidney failure, cardiovascular death, or kidney death overall and by the individual components using laboratory data for eGFR results

Cardiorenal Composite and Components	Sotagliflozin (*N*=5292), *n* (%)	Placebo (*N*=5292), *n* (%)	HR (95% CI)	*P* Value
**First event in composite of 50% decline in eGFR** **(sustained or last value), kidney failure^a^,** **CV death, or kidney death**	239 (4.5)	306 (5.8)	0.77 (0.65 to 0.91)	0.002
50% decline in eGFR	75 (1.4)	121 (2.3)	0.60 (0.45 to 0.81)	<0.001
Kidney failure	50 (0.9)	78 (1.5)	0.63 (0.45 to 0.91)	0.01
*eGFR <15 ml/min per m*^*2*^	41 (0.8)	68 (1.3)	0.60 (0.41 to 0.88)	0.009
*Maintenance dialysis or kidney transplant*	15 (0.3)	22 (0.4)	0.67 (0.35 to 1.30)	0.24
CV death	155 (2.9)	170 (3.2)	0.90 (0.73 to 1.12)	0.36
Kidney death	8 (0.2)	5 (0.1)	1.59 (0.52 to 4.86)	0.66

CI, confidence interval; CV, cardiovascular; HR, hazard ratio.

a Kidney failure was defined as eGFR <15 ml/min per m^2^ (sustained or last value), kidney transplant, or initiation and maintenance of dialysis.

**Figure 2:**
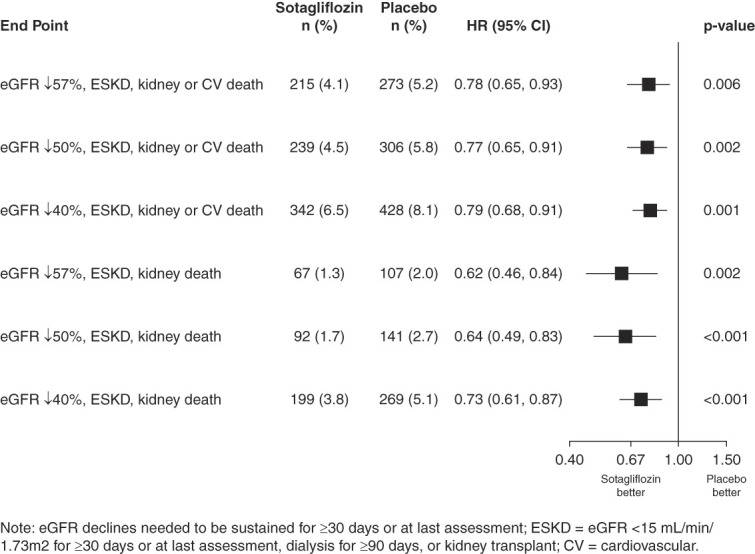
**Forest plot of incidence of various kidney and cardiorenal composites by treatment groups using laboratory data for eGFR results.** CV, cardiovascular.

In additional analyses, the results were generally consistent when using different eGFR decline thresholds and/or evaluating kidney-related outcomes (all *P* < 0.01, Figure 2).

### Subgroup Analyses

**Figure 3:**
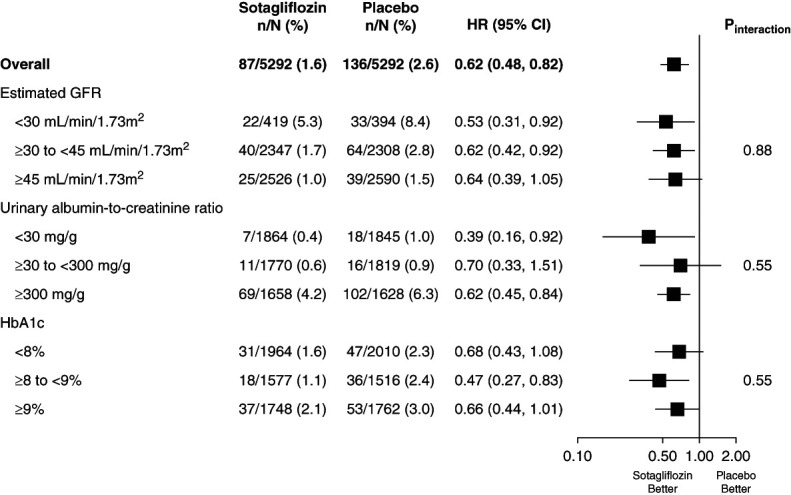
**Forest plot with subgroups for the composite of first event of 50% decline in eGFR or kidney failure defined as eGFR <15 ml/min per 1.73 m**^**2**^**, maintenance dialysis, or kidney transplant using laboratory data for eGFR results.** HbA1c, hemoglobin A1c.

The effect of sotagliflozin versus placebo for the composite outcome of ≥50% decline in eGFR or kidney failure was consistent when evaluated by subgroups of baseline eGFR, HbA1c, and UACR (*P* > 0.5 for interaction; Figure 3).

To take advantage of the larger event numbers, the same subgroups were examined for the composite outcome of ≥50% decline in eGFR, kidney failure, CV death, or kidney death. The effects of sotagliflozin were consistent when evaluated by subgroups of baseline eGFR and HbA1c (*P* > 0.2 for interaction), but varied by baseline UACR, with the largest effect in patients with baseline UACR ≥300 mg/g and neutral effects in people with UACR <30 or 30–300 mg/g (*P* value for interaction = 0.02; Supplemental Figure 2).

### Sotagliflozin and AKI

AKI events were lower in the sotagliflozin group (6%; 369 events in 300 patients) versus the placebo group (7%; 442 events in 346 patients) (relative risk [95% CI], 0.90 [0.70 to 1.00]). In both the sotagliflozin and placebo groups, the incidence of serious AKI events (1.7% in both groups) and AKI events leading to permanent treatment discontinuation (0.4% and 0.5%, respectively) was low. In both treatment groups, AKI was reported more frequently in patients with baseline eGFR <30 ml/min per 1.73 m^2^ compared with those with baseline eGFR ≥30 ml/min per 1.73 m^2^ and in men compared with women (Supplemental Table 1).

## Discussion

In the original SCORED analysis, the predefined kidney-specific composite end point was reduced by 29% with sotagliflozin compared with placebo, a difference that did not reach statistical significance. The early study termination and lack of reconciliation of eGFR-based end points with case report form and laboratory data limited the number of events captured and, as a result, the statistical power to detect a difference. This exploratory analysis set to examine the totality of eGFR-based end points available from laboratory data. This approach yielded substantially more events and showed a large (38%) and statistically significant reduction in this predefined end point with sotagliflozin compared with placebo. The original SCORED report only showed a single kidney composite end point as part of the statistical hierarchy. Although several definitions of kidney-specific and cardiorenal composite end points have been evaluated in SGLT2 inhibitor trials, this exploratory analysis focused on a composite outcome using a first event of 50% decline in eGFR, kidney failure, CV death, or kidney death and demonstrated that sotagliflozin reduced the risk of this end point by 23% compared with placebo in patients with type 2 diabetes and CKD, suggesting a kidney protective effect with sotagliflozin in patients with type 2 diabetes and CKD.

Kidney protection with sotagliflozin is consistent with the results of other kidney outcome trials with selective SGLT2 inhibitors, including Canagliflozin and Renal Events in Diabetes with Established Nephropathy Clinical Evaluation (CREDENCE), The Study to Evaluate the Effect of Dapagliflozin on Renal Outcomes and Cardiovascular Mortality in Patients with CKD (DAPA-CKD), and Empagliflozin in Patients with Chronic Kidney Disease (EMPA-KIDNEY).^13–15^ There have been differences in the definitions for kidney and cardiorenal composite end points used in kidney outcome trials, as well as in the prespecified kidney analyses of CV outcomes trials of SGLT2 inhibitors. These variable definitions make cross-study comparisons difficult. Statistically and clinically significant evidence of kidney protection was observed with sotagliflozin across all commonly used kidney and cardiorenal composite end points, including those outcomes incorporating ≥57% decline in eGFR as part of the definition of the composite.^13^ The ability to detect larger declines in kidney function generally requires cohorts at higher risk of CKD progression, with longer periods of observation, or with a larger treatment effect. On the basis of laboratory-based outcomes, kidney benefits with sotagliflozin were observed despite a median follow-up of only 16 months in the SCORED trial. Furthermore, participants enrolled in the SCORED trial were likely a lower kidney risk cohort than the three other dedicated kidney outcome trials because of the different entry criteria, including any level of albuminuria that yielded lower median baseline UACR and percentage of patients with severely increased albuminuria. The results of this analysis, therefore, suggest that sotagliflozin confers kidney protection that is similar to the effects attributed to selective SGLT2 inhibitors, in keeping with a consistent cardiorenal protection across different agents in this class including patients with a spectrum of kidney and CV risk.^2,16^

Kidney protective effects of sotagliflozin were not modified by baseline glycemic control, consistent with the known glucose-independent effects of SGLT inhibition.^17–20^ There was also no evidence of heterogeneity across baseline kidney function, including in participants with eGFR ≤30 ml/min per 1.73 m^2^. Our results are consistent with other analyses in the CREDENCE, DAPA-CKD, and EMPA-KIDNEY trials demonstrating efficacy in patients with eGFR <30 ml/min per 1.73 m^2^.^15,21,22^ Conversely, and in line with previous SGLT inhibitor trials, we did observe effect modification by baseline level of albuminuria, as demonstrated by a larger risk reduction for the cardiorenal composite end point among participants with severely increased albuminuria compared with those with A1 or A2 albuminuria. These results differ from those reported in similar analyses of the CREDENCE and DAPA-CKD trials,^21,22^ although these trials excluded patients with A1 albuminuria, and the CREDENCE trial had small numbers of patients with UACR in the A2 range and also excluded individuals with eGFR <30 ml/min per 1.73 m^2^. Nevertheless, the statistical heterogeneity across UACR levels is consistent with effect modification reported by baseline UACR in the EMPA-KIDNEY trial, reflecting higher event rates in participants with severely increased albuminuria. There was, however, no evidence of effect modification by baseline albuminuria when evaluating the prespecified kidney composite outcome of ≥50% decline in eGFR or kidney failure.

In addition to benefits around efficacy with sotagliflozin as described in this exploratory analysis, sotagliflozin was also associated with a numerical reduction in the risk of AKI compared with placebo. The reduction in AKI risk with SGLT2 inhibitors is well described in both clinical trials with real-world analyses and may be on the basis of several mechanisms, including a reduction in the risk of kidney ischemia.^23–27^ Regardless of the responsible pathways, SGLT inhibitors appear to be distinct compared with other kidney protective therapies, such as renin-angiotensin system inhibitors, in that the well-described acute and reversible hemodynamic effects of SGLT inhibitors are associated with long-term benefits and consistently reduce AKI risk by 15%–20% in patient with and without diabetes.^2^

There are important limitations to our study. First, this was a *post hoc* analysis—while we used the predefined kidney-specific composite outcomes from the primary report from the SCORED trial, we ascertained eGFR-based kidney events using laboratory data. However, this was performed while blind to treatment allocation. Second, we did not perform an adjudication of the original case report form–based kidney composite reported in the primary report of the SCORED trial and cannot determine whether the currently reported laboratory-derived eGFR kidney events agreed with the case report form–derived eGFR kidney events. Nonetheless, laboratory data provide an objective and unbiased approach to ascertain outcomes. The compatible findings in our analysis and the SCORED primary report provide strong evidence of the kidney protective benefit of sotagliflozin. In addition, the consistent findings among the various kidney-specific and cardiorenal composite end points in this analysis and compared with results with SGLT2 inhibitors in other CKD studies support a class effect on kidney outcomes. Finally, subgroup analyses are hypothesis generating and not powered to detect treatment effects within the evaluated subgroups.

In conclusion, in this analysis of the SCORED trial, sotagliflozin substantially and significantly reduced rates of CKD progression. The results were consistent when using different eGFR decline thresholds and across baseline eGFR and glycemic control subgroups. Kidney protection with sotagliflozin is consistent with known benefits of selective SGLT2 inhibitors. This benefit is in addition to the significant reduction in heart failure events seen with sotagliflozin and other SGLT inhibitors, as well as the significant reduction in major adverse CV events seen with sotagliflozin,^4^ although not consistently seen with the more selective SGLT2 inhibitors in the CKD trials.

## Supplementary Material

## Data Availability

Data may be shared on reasonable request to the corresponding author.

## Supplemental Material

Supplemental Table 1. Overview of AKI—safety population.

Supplemental Figure 1. Cumulative incidence curve for the composite of first event of 50% decline in eGFR, ESKD, CV death, or kidney death.

Supplemental Figure 2. Forest plot with subgroups for the composite of first event of 50% decline in eGFR, ESKD, CV death, or kidney death using laboratory data for eGFR results.
