# Supplementary material for: Sotagliflozin and Kidney Outcomes, Kidney Function, and Albuminuria in Type 2 Diabetes and CKD: A Secondary Analysis of the SCORED Trial
Source: Clin J Am Soc Nephrol. 2024 Jan 26;19(5):557–64. doi: 10.2215/CJN.0000000000000414 (PMC11108248; doi:10.2215/CJN.0000000000000414)

Supplemental Materials (Sridhar, Bhatt, Odutayo et al.)

Effects of sotagliflozin on kidney composite outcomes, kidney function, and albuminuria in patients with type 2 diabetes and chronic kidney disease: a comprehensive analysis of the SCORED trial

Vikas S. Sridhar, MD, Deepak L. Bhatt, MD, MPH, Ayodele Odutayo, MD, DPhil, Michael Szarek, PhD, Michael J. Davies, PhD, Phillip Banks, MS, Bertram Pitt, MD, Ph. Gabriel Steg, MD, David Z. I. Cherney, MD, PhD

Table of Contents

Supplemental Table 1:	Overview of acute kidney injury – Safety Population	3
Supplemental Figure 1.	Cumulative incidence curve for the composite of first event of 50% decline in eGFR, ESKD, CV death, or renal death	5
Supplemental Figure 2.	Forest plot with subgroups for composite of first event of 50% decline in eGFR, ESKD, CV death, or renal death using laboratory data for eGFR results	6

Supplemental Table 1: Overview of acute kidney injury – Safety Population

	**Sotagliflozin (N=5291)**	**Placebo (N=5286)**
Total exposure (patient-years)	6385.1	6369.7
Number of patients with events, n (%)	300 (5.7)	346 (6.5)
Number of patients with events per 100 patient-years [a]	4.7	5.4
Number of events	369	442
Number of events per 100 patient-years [b]	5.8	6.9
Relative risk (95% CI vs placebo)	0.9 (0.7, 1.0)	
Any event considered serious, n (%)		
Yes	91 (1.7)	92 (1.7)
No	163 (3.1)	191 (3.6)
Any event considered IMP related, n (%)		
Yes	50 (0.9)	54 (1.0)
No	204 (3.9)	229 (4.3)
Any event led to permanent discontinuation of IMP, n (%)		
Yes	20 (0.4)	29 (0.5)
No	234 (4.4)	254 (4.8)
Worst severity, n (%)		
Mild	66 (1.2)	76 (1.4)
Moderate	122 (2.3)	142 (2.7)
Severe	66 (1.2)	64 (1.2)
Age group 1 (years) [c]		
<65	85/1582 (5.4)	129/1640 (7.9)
≥65	215/3709 (5.8)	217/3646 (6.0)
Age group 2 (years) [c]		
<75	227/4051 (5.6)	272/4078 (6.7)
≥75	73/1240 (5.9)	74/1208 (6.1)
Sex[c]		
Female	111/2347 (4.7)	118/2406 (4.9)
Male	189/2944 (6.4)	228/2880 (7.9)
Baseline BMI (kg/m^2) [c]		
<30	112/1934 (5.8)	136/1981 (6.9)
≥30	188/3356 (5.6)	210/3304 (6.4)
Baseline eGFR (mL/min/1.73m^2) [c]		
<30	36/418 (8.6)	34/393 (8.7)
≥30 to <45	143/2346 (6.1)	157/2305 (6.8)
≥45	121/2526 (4.8)	155/2587 (6.0)

	Sotagliflozin (N=5291)	Placebo (N=5286)
Baseline LVEF (%) [c]		
<50	71/1077 (6.6)	81/1082 (7.5)
≥50	228/4202 (5.4)	265/4199 (6.3)

CI = confidence interval; IMP = investigational medicinal product; vs = versus.
[a] Calculated as number of patients with events divided by total exposure in 100 patient-years.
[b] Calculated as number of events divided by total exposure in 100 patient-years.
[c] For the subgroup categories, % based upon n/N, where N=number of patients in the subgroup for the treatment regardless of whether they had event.
Patient-years of exposure: calculated as time from the first to the last administration of IMP plus 1 day, divided by 365.25.
Relative risk is the ratio of the risk of having an acute kidney injury-specified event in the sotagliflozin group compared to the risk of having the event in the placebo group.
Treatment-emergent events included are those with an onset after the first IMP dose until 10 days after the last IMP dose.
n (%) = number and percentage of patients with at least one occurrence of the event where percentages are calculated using the number in each treatment group in the safety population as the denominator.

Supplemental Figure 1. Cumulative incidence curve for the composite of first event of 50% decline in eGFR, ESKD, CV death, or renal death

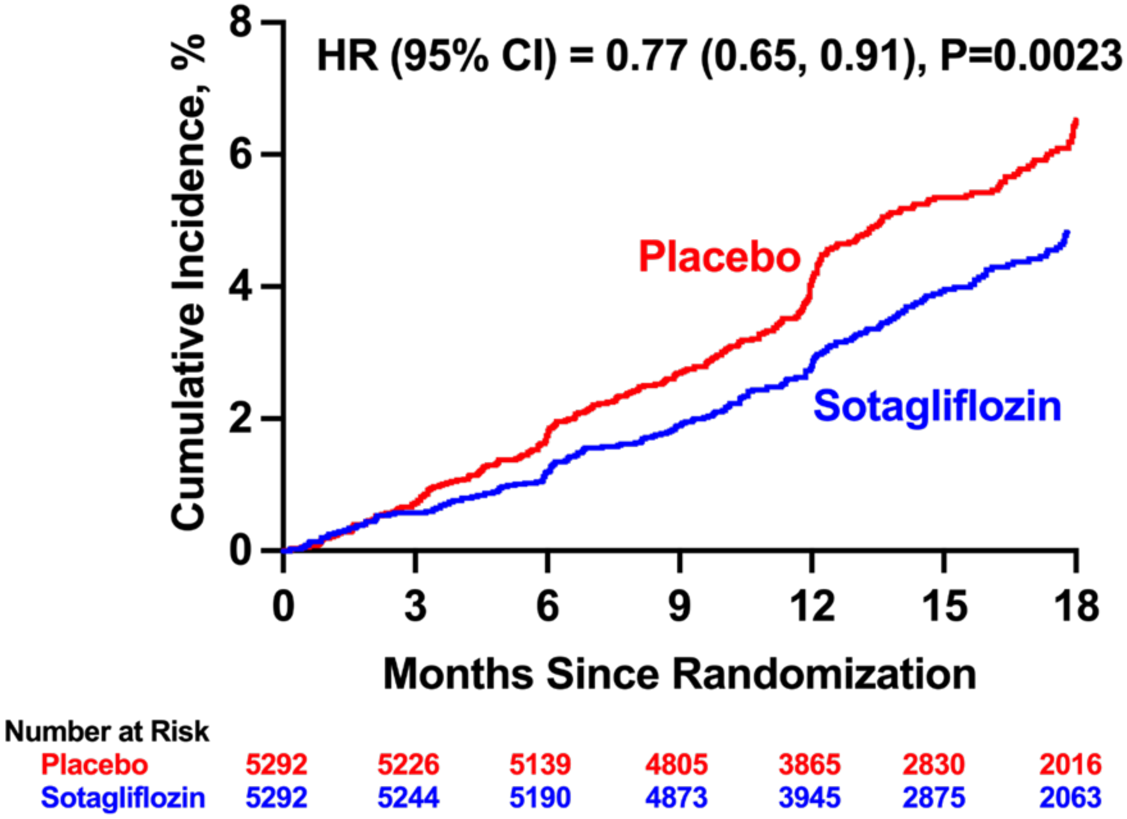

Supplemental Figure 2. Forest plot with subgroups for composite of first event of 50% decline in eGFR, ESKD, CV death, or renal death using laboratory data for eGFR results

	Sotagliflozin n/N (%)	Placebo n/N (%)	HR (95% CI)	$P_{interaction}$
**Overall**	**239/5292 (4.5)**	**306/5292 (5.8)**	**0.77 (0.65, 0.91)**	
Estimated GFR				
<30 mL/min/1.73m^2	42/419 (10.0)	50/394 (12.7)	0.72 (0.48, 1.09)	
≥30 to <45 mL/min/1.73m^2	112/2347 (4.8)	163/2308 (7.1)	0.68 (0.53, 0.86)	0.2906
≥45 mL/min/1.73m^2	85/2526 (3.4)	93/2590 (3.6)	0.92 (0.68, 1.23)	
Urinary albumin-to-creatine ratio				
<30	45/1864 (2.4)	50/1845 (2.7)	0.90 (0.60, 1.35)	
≥30 to <300	80/1770 (4.5)	78/1819 (4.3)	1.03 (0.75, 1.41)	0.0170
≥300	114/1658 (6.9)	178/1628 (10.9)	0.60 (0.47, 0.76)	
HbA1c				
<8%	83/1964 (4.2)	104/2010 (5.2)	0.82 (0.61, 1.09)	
≥8 to <9%	61/1577 (3.9)	77/1516 (5.1)	0.77 (0.55, 1.08)	0.7969
≥9%	94/1748 (5.4)	125/1762 (7.1)	0.72 (0.55, 0.94)	

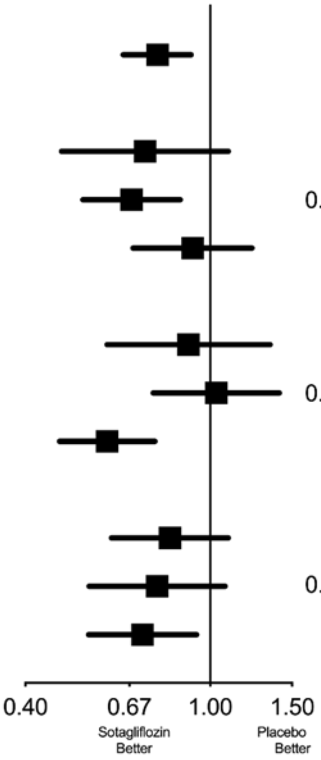

Supplement: Supplementary file 1 [file cjasn-19-557-s001.pdf]
